# Iodine deficiency and its consequences for cognitive and psychomotor development of children

**DOI:** 10.1186/1824-7288-40-S1-A15

**Published:** 2014-08-11

**Authors:** Roberto Gastaldi, Monica Muraca, Agnese Beltramo, Elena Poggi

**Affiliations:** 1 IRCCS Giannina Gaslini, Genoa, Italy; 2 Pediatric Department University of Genoa, Genoa, Italy

## 

Dietary iodine intake is required for the production of thyroid hormones. Thyroxine (T4) and triiodothyronine (T3) are essential for the development of the central nervous system, they regulate genes involved in myelination and neuronal/glial cell differentiation, and play an important role in axonal and dendrite growth in synaptogenesis and myelination. Deficiencies in thyroid hormones in pregnant women directly affect brain development of the fetus, resulting in neurological and neurocognitive disorders in infants with defects ranging from decrease in intelligence and lethargy to mental retardation. Thyroid deficiency at different stages of pregnancy affects different brain regions, for example, basal ganglia are affected by early thyroid hormone deficiency and cerebellar and hippocampal development is influenced by late thyroid dysfunction [1]. Therefore the consequences of the brain damage depend upon the timing and severity of the hypothyroxinaemia, when it appearing before or at 12 weeks of pregnancy it may affect cognitive development. The neurological cretinism, a disease characterized by severe neurological lesions without clinical hypothyroidism may be a consequence of severe hypothyroxinaemia in early pregnancy. Congenital hypothyroidism is one of the most common preventable causes of mental retardation in children and describes the deficiency in thyroid hormones as of prenatal onset of severe thyroid dysfunction. Newborn screening and thyroid therapy started within 2 weeks of age can normalize cognitive development. Mild reductions in maternal thyroid hormone levels in early pregnancy are associated with reduced IQ in offspring and prenatal exposure to maternal hypothyroxinaemia increased the risk of expressive language delay and nonverbal cognitive delay in preschool age children [2]. Mild iodine deficiency during pregnancy may lead to hypothyroxinaemia in the mother and/or elevated thyroid-stimulating hormone levels in the foetus, and these conditions have been found to be related to mild and subclinical cognitive and psychomotor deficits in neonates, infants and children [3]. Moderate cognitive impairments have been observed in children from the age of 3 weeks up to 5 years. Low maternal iodine status could be associated with an increased risk of suboptimal scores for verbal IQ at age 8 years and reading accuracy, comprehension and reading scores at age 9 years. Correction of mild-to-moderate iodine deficiency improves cognitive performance in school age children [4]. As iodine deficiency is still the most widespread cause of maternal hypothyroxinaemia, the birth of many children with learning disabilities may already be preventable by advising women to take iodine supplements as soon as pregnancy starts, or earlier if possible.
